# Insights and perspectives: EUS in post-liver transplantation care

**DOI:** 10.1097/eus.0000000000000084

**Published:** 2024-11-05

**Authors:** Eyad Gadour, Bogdan Miutescu, Hussein Hassan Okasha

**Affiliations:** 1 Department of Gastroenterology and Hepatology, King Abdulaziz National Guard Hospital, Ahsa, Eastern Region 36248, Saudi Arabia; 2 Department of Medicine, Faculty of Medicine, Zamzam University College, Khartoum 11113, Sudan; 3 Department of Gastroenterology and Hepatology, Victor Babeş University of Medicine and Pharmacy, Timisoara 300041, Romania; 4 Department of Internal Medicine, Division of Gastroenterology and Hepatology, Kasr Al-Aini School of Medicine, Cairo University, Cairo 11562, Egypt.

## INTRODUCTION

Liver transplant (LT) is a therapy that has rapidly evolved since it was first successfully performed in 1963 by Thomas Starzl in the United States.^[1]^ Currently, liver transplant is the recommended standard therapy for acute liver failure and end-stage liver disease, regardless of the etiology.^[2]^ With the increased number of liver transplants being performed over the years, the outcomes of most patients with liver failure have improved significantly.^[2]^ For instance, in Europe, over the past decades, the 1-year survival rate of posttransplant patients has increased to 83%, the 5-year survival rate to 71%, and the 10-year survival rate to 61%.^[3]^ This success of liver transplants has been greatly influenced by various factors, such as the development of immunosuppressive agents, improved storage facilities, and better surgical techniques during liver transplantations.^[4]^ Another attribute ensuring this success is improved management of post-liver transplant complications, although more is yet to be achieved in this area.

Even with technological improvements, the rate of complications in post-liver transplant patients is still very high and a leading cause of late mortality in these patients.^[5]^ For instance, even with the uptake of immunosuppressive therapy in post-liver transplant patients, immunosuppressive treatment has been linked to other complications that increase the risk of mortality in posttransplant patients.^[6]^ These complications include cardiovascular events, malignancy, allograft failure, renal failure, and infections.^[5]^ Other complications faced by liver transplant patients include biliary complications, vascular complications, parenchymal complications, and small-for-size syndrome (SFSS).^[7–9]^ In managing the complications that present after a liver transplant, patient triaging is a very crucial step. This step involves the clinician using his clinical expertise to determine the diagnostic tests to administer to the patients according to their clinical presentation and posttransplant presentation time. For example, suppose a patient presents with abnormal liver function tests within 2 weeks posttransplant. In that case, they are more likely to have portal vein or hepatic artery thrombosis than hepatic vein stenosis.^[9]^ Furthermore, knowledge of the basic imaging algorithm for posttransplant patients is crucial in triaging. The initial imaging performed during triaging is typically evaluation with Doppler ultrasonography.^[10]^ Furthermore, diagnostic ERCP (D-ERCP) is also used to assess cases of suspected biliary complications that do not meet the criteria for therapeutic interventions.^[11]^ If the initial imaging is normal, a biopsy is conducted to rule out allograft rejection or other etiologies of parenchymal disease.^[9]^ If the imaging detects any complication, they are further evaluated using catheter angiography, computed tomography (CT), or magnetic resonance imaging (MRI).^[9,10]^ In addition to these conventional diagnostic tools, endoscopic ultrasound (EUS) has also been used to diagnose some of the complications.

EUS combines 2 imaging modalities: endoscopic visualization and high-frequency ultrasound imaging.^[12]^ This novel imaging technique was developed in the 1980s and is widely used for its advantage of providing real-time imaging of all the layers of the gastrointestinal wall and extending further beyond into the vessels and organs in proximity.^[13]^ Furthermore, the combination of EUS with other diagnostic tools such as fine needle aspiration (EUS-FNA) and fine needle biopsy (EUS-FNB) has enabled the access of previously difficult-to-access anatomic locations and enabled the obtaining of tissue samples for histological diagnosis of some pathologies.^[14]^ Unlike conventional diagnostic tools, EUS is an emerging tool for diagnosing complications in post-liver transplant patients whose wide adoption is yet to be achieved. EUS has been used mainly to diagnose biliary complications in post-liver transplant transplants.^[15]^ Furthermore, EUS-FNA has enabled obtaining tissue and fluid samples for analysis in the confirmative diagnosis of some malignancies and other complications. Lastly, apart from diagnosis, EUS is also used to guide other therapeutic interventions used to manage post-liver transplant complications.

## ROLE OF EUS IN SURVEILLANCE OF POSTTRANSPLANT COMPLICATIONS

EUS achieves very high-resolution and clear images of the bile duct gastrointestinal anatomies and has thus evolved to become one of the diagnostic tools for biliary obstruction over the past decades. In non-liver transplant settings, EUS has demonstrated high diagnostic specificity and sensitivity for biliary disorders.^[16,17]^ In transplant settings, however, there is limited research on the application of EUS in diagnosing post-liver transplant biliary complications. It has been mainly used in post-liver transplant patients to diagnose biliary tree and parenchymal complications. Furthermore, it can aid in the histological diagnosis of parenchymal disease or malignancy in post-liver transplant patients when combined with other diagnostic modalities such as fine needle aspiration and fine needle biopsy.^[18]^ EUS is one of the imaging studies carried out on post-liver transplant patients who present with abnormal liver function tests (LFTs) within a year of liver transplant.^[18]^ Because some post-liver transplant patients present with nondiagnostic abnormal LFTs, it is necessary to establish a precise sequence of appropriate tests for definitive diagnosis. In this cohort of patients, a pilot cohort study has demonstrated the feasibility of EUS-guided liver biopsy and D-ERCP offered in a single session to enhance patient outcomes. The use of both these procedures enables the diagnosis of multiple post-liver transplant complications, such as anastomotic strictures and T-cell–mediated rejection of the allograft.^[18]^

Empirical evidence on the benefits and advantages of using EUS in diagnosing posttransplant complications is yet to be reported. It is, however, scientifically sound to infer that the procedural advantages of EUS diagnosis of liver pathologies in other settings will be similar to those in post-liver transplant settings. The benefits of using EUS in diagnosing liver complications may include the following: EUS imaging, being a sedated procedure, is more comfortable for the patients.^[19]^ Furthermore, providing real-time imaging and ultrasound guidance during the procedure allows the endoscopist to visualize all the large vessels and organs. It thus minimizes the risk of complications such as bleeding.^[19]^

## DIAGNOSTIC ACCURACY OF EUS IN DIFFERENTIATING POSTTRANSPLANT COMPLICATIONS FROM COMMON POSTOPERATIVE CHANGES

The gold standard for imaging in post-liver transplant patients is D-ERCP. However, endoscopic ultrasound has been an emerging diagnostic tool with almost similar efficacy in diagnosing post-liver transplant patients’ complications. In managing biliary complications in post-liver transplant patients, EUS demonstrated an overall accuracy, specificity, and sensitivity of 94.6%, 100%, and 94.6%, respectively.^[15]^ When the individual biliary complications are considered, EUS has different diagnostic capabilities, varying according to the complications. For instance, EUS has a sensitivity of 100% in diagnosing biliary casts, ischemic cholangiopathy, and ischemic type biliary lesions (ITBL), whereas it has a sensitivity of 88.2% in diagnosing anastomotic strictures.^[15]^

Although liver transplant is the definitive treatment of most malignancies of the liver, such as hepatocellular carcinoma (HCC), the rate of reoccurrence of these malignancies is very high, about 25% within the first 2 years.^[20]^ With this high recurrence rate, frequent imaging is necessary to assess for recurrence of the HCC in these patients. EUS-FNA has, therefore, been applied in some cases to aid in the diagnosis of recurrence. For instance, EUS-FNA has been successfully used in diagnosing intrahepatic reoccurrence of HCC in post-liver transplant patients.^[21]^ Apart from recurring neoplasms, EUS-FNA has also been crucial in the confirmatory diagnosis of some benign lesions, such as schwannoma, in post-liver transplant patients.^[22]^ Using EUS-FNA to diagnose these lesions enables confirmative diagnosis based on the imaging and histological findings of the tissue sample obtained.

## THERAPEUTIC APPLICATIONS OF EUS-GUIDED THERAPIES

Over the past decades, EUS has increased its application beyond diagnosis and enabled various guided therapeutic interventions that are minimally invasive. In post-liver transplant settings, however, little evidence has indicated the frequent use of EUS-guided therapeutic interventions. Only a few case reports have reported the feasibility of EUS-guided therapeutic interventions in managing post-liver transplant complications. The instances where EUS-guided therapies have been successfully used to manage post-liver transplant complications include the following: In patients with malignant distal biliary obstruction, EUS-guided cholangiopancreatoscopy with LAMS has been used to enable the placement of lumen-apposing stents (LAMS) for definitive treatment of recurrent cholangitis after T-ERCP.^[23]^ In patients with post-liver transplant ascites, EUS-guided drainage has been successfully carried out and induced complete resolution of the symptoms. However, these patients required several sessions of EUS-guided drainage to resolve symptoms fully.^[24]^ This need for extra sessions for the complete resolution of symptoms indicates that EUS-guided drainage may not be very efficacious in managing these symptoms. In patients with post-liver transplant biliary casts, EUS has been used to guide alteration of the upper gastrointestinal anatomy, i.e., create an endoscopic gastrojejunostomy using LAMS. This enabled easy access to the billioenteric anastomoses, thus allowing the performance of other complex biliary interventions to manage complicated biliary pathologies such as biliary casts.^[25]^

From the few case reports identified, it is observed that EUS-guided ostomy procedures with placement of LAMS are feasible and crucial in managing some recurrent complications that are not fully treated using conventional ERCP treatments. Furthermore, it is noted that EUS-guided therapies have been proposed to minimize the risk of infections and bleeding compared to other interventions, such as surgery.^[26]^

## EFFICACY OF EUS COMPARED TO OTHER IMAGING STUDIES

Compared to D-ERCP, the overall efficacy of EUS in diagnosing biliary complications is inferior. For instance, the sensitivity and accuracy of D-ERCP in diagnosing biliary complications are 97.3% compared to 94.6% of EUS.^[15]^ However, both methods have a specificity of 100% in diagnosing biliary complications.^[15]^ Although D-ERCP is inherently superior to EUS in diagnosing biliary complications, some of its procedural factors limit its efficacy in diagnosing some biliary complications. For example, the sensitivity of EUS in diagnosing biliary casts was superior to D-ERCP (100% compared to 80%) partly because the contrast administered into the bile duct during D-ERCP masks the casts.^[15]^ EUS has not been compared to other diagnostic tools in post-liver transplant patients. Furthermore, the comparative observational study only analyzed the efficacy of EUS in diagnosing biliary complications; the comparative efficacy in diagnosing the other complications still needs to be established.

## LIMITATIONS OF THE USE OF EUS IN MANAGING POST-LIVER TRANSPLANT PATIENTS

The limitations of EUS in post-liver transplant patients are not well established. This is because most population-based cohort studies have been based on most conditions’ pretransplant, and only one study has compared it to D-ERCP at a population level. With the few case reports that have demonstrated its use in post-liver transplant, it is not possible to infer meaningful conclusions based on the reported results due to potential publication bias and selective reporting of the observed results. Nevertheless, a few challenges have been reported in using EUS in post-liver transplant. In a case series by Uchida et al.,^[24]^ it was noted that some patients who underwent EUS-guided ascites drainage developed procedure-related complications such as bleeding, peritonitis, and stent migrations. Another challenge is the limited adoption of EUS in managing complications in post-liver transplant patients. This is because the overall efficacy of EUS is inferior to some conventional diagnostic tools such as D-ERCP.

Even though EUS is inferior to D-ERCP in diagnosing post-liver transplant complications, in some instances, EUS has been reported to aid in treating patients who had received prior failed T-ERCP sessions. Therefore, the clinical significance of EUS in post-liver transplant patients cannot be overlooked. We thus make the following recommendations for future research on EUS application in post-liver transplant patients.

1. Observational studies should be conducted to assess the efficacy of EUS compared to conventional diagnostic tools such as ERCP in diagnosing post-liver transplant complications in liver transplant patients.
2. Studies should be conducted to determine the feasibility of EUS-guided therapies in managing post-liver transplant complications. These therapies include EUS-guided ascites drainage and EUS-guided ostomy procedures to manage various complications that occur in patients with altered gastrointestinal anatomy.
3. Future trials and observational studies should be carried out to assess the efficacy of EUS-FNA in diagnosing some post-liver transplant complications, such as recurrent hepatocellular carcinoma and parenchymal liver complications.
4. The studies should also assess the safety profile of EUS-guided therapies and EUS-FNA in post-liver transplant patients because these patients are predisposed to developing infectious complications due to long-term immunosuppressive therapy.

## CONCLUSION

Based on current evidence, it is clear that the use of EUS in post-liver transplant patients is not extensively adapted in most settings. However, its current applications in the surveillance of post-liver transplant complications are mainly rooted in diagnosing biliary complications. EUS diagnostic capabilities in diagnosing post-liver transplant biliary complications are promising and similar to conventional diagnostic tools such as ERCP. Furthermore, when combined with diagnostic tools such as FNA, EUS-FNA has proven to be a crucial complementary tool in ensuring early diagnosis of post-liver transplant complications that routine ERCP cannot diagnose.

Apart from diagnosis, some empirical evidence has ascertained the feasibility of therapeutic application of EUS-guided procedures in managing some posttransplant procedures. Specifically, the use of EUS-guided ostomy procedures in managing some biliary complications accompanied by altered gastrointestinal anatomy has been reported on some occasions. However, the broad adoption of EUS-guided procedures in managing most post-liver transplant complications is yet to be done.
